# The Dynamics of Organic Life

**Published:** 1869-04-15

**Authors:** Z. C. M’Elroy

**Affiliations:** Zanesville, Ohio


### CORRESPONDENCE.

### *The Dynamics of Organic Life.*

BY Z. C. M'ELROY, M.D., ZANESVILLE, OHIO.

No author should ever feel displeased with such a notice of his labors as you were pleased to make in The Journal for the first March, ult., of my monograph "On the Dynamics, Principles and Philosophy of Organic Life."

The question of most interest connected with my proposal to regard all diseased action as modifications of the organic processes of constructive and destructive metamorphosis, and to classify all therapeutic agents as they are found, by experience, to promote or retard them, is, undoubtedly, "How will they work practically?"

The same number of THE JOURNAL contains the conclusion of a very interesting paper by Dr. Wallihan, of Chicago, "On Super Oxygenation as a Therapeutic Measure, with Notes of Cases Treated." These cases it is proposed to take as examples, for the purpose of ascertaining whether they can be viewed as lesions of nutrition and oxidation, and if the treatment pursued influenced these processes, by either promoting or retarding them; and what were the relations of the pathological conditions, and therapeutic measures employed, to the living organism.

As at once a base and starting point in the investigation the following propositions are submitted:

1st.—A change of matter must occur, simultaneously with every manifestation of force, whether in the organic or inorganic kingdoms of nature.

This proposition, it is believed, is universally assented to by the philosophic and scientific world, and, therefore, needs neither proofs nor illustrations.

2d.—There is neither local disease or local remedy.

This is, perhaps, not assented to by any considerable portion of the medical world. It is so contrary to the teachings of the past, as well as the current ideas of the present, that it may require both illustration and proof. The dogma of "the indestructibility of matter" was the basis from which chemistry leaped into an exact science. "The persistence of force" is the magic formula from which rises exact natural science, and "that there is neither local disease or local remedy," is the basis from which will grow an exact science of therapeutics and practical medicine. And it can grow from no other. That disease does have local manifestations is certainly true; but the generic totals of constructive and destructive metamorphosis must first undergo modifications. So long as these proceed normally in the individual organism, the result is health, not disease—harmony, ease, not discord or dis-ease. Take the alphabetical catalogue of diseases, examine each separately, from "abortion and abscess," to "wounds, and zona, argenti nitratis," and it will be found that from the slightest head-ache, to the gravest form of fever, the organic processes of nutrition and oxidation are constantly interfered with; and in proportion to the extent of the interference is the gravity of the diseased action. Regard them as separate entities, and the confusion of therapeutics in the past reigns supreme in the future.

But the limits proposed for this paper will not permit of further proof. It may not be new to either editors or readers of THE JOURNAL, but it is new and original with the writer. It underlies the whole contents of my monograph —forms the only safe foundation for a science of therapeutics worthy of the name, as well as a science of pathology; or, for that matter, a science of life—Biology.

Dr. Wallihan has been treating certain cases of disease, by introducing into the system a greater proportion of oxygen than is contained in the atmosphere, and has found certain forms of chronic disease to be greatly benefitted. In the absence of any scientific generalization of the relations of oxygen, as well as disease, to the organic processes of life, he wisely feels his way, by carefully collecting the reported results of its empirical employment, as well as the mode and form in which it has been employed by others in the past. Far be it from me to find any fault with Dr. Wallihan, in the course he has pursued. He has worked with accredited agents, and in the accredited modes of the profession, and has done a good work, for which he deserves, and no doubt will receive, the thanks of your readers and the profession. His paper very properly winds up with reports of some of his cases, the treatment consisting in simply introducing into his patients' systems an excess of oxygen over that contained in the atmosphere which all breathe.

What does oxygen do in the laboratory of organic life? Beyond all cavil its mission is double—death and life. It is the most potent agent in nature, "promoting destructive metamorphosis." And, as the dynamics of all organic life depend on changes of matter, the surplus oxygen he introduced into his patients' systems added something to the sum of the forces of life. He increased activity in the process of destructive metamorphosis, liberated the necessary dynamic force for "promoting constructive metamorphosis," *i. e.*—death and life. In due time, by thus promoting both destructive metamorphosis and constructive metamorphosis—if he gave his patients the necessary organic material in the shape of good food—the normal velocity of tissue change was restored, and the results, his patients were well.

But examine his cases separately, and more in detail, and see whether, pathologically, the organic processes of nutrition and oxidation were proceeding normally.

Case 1st, presents a patient in whom, certainly, the processes of "constructive and destructive metamorphosis" are sadly deranged. Nutrition is capricious, and waste unequal. The normal velocity of tissue changes are widely departed from. She is what is called "hysterical;" that is, her dynamic harmony has been broken up. But there are no marked structural lesions; they are wholly confined to "constructive and destructive metamorphosis." She probably belonged among "well-to-do people," and did not perform the requisite physical labor to make good her daily normal tissue-waste. No mention is made of summing up the symptoms of the temperature in her axilla, which is to be regreted, as the results of a few days' observation would have thrown much light on her case. Theoretically, it should have been variable, before treatment, alternately above and below the normal standard; while, after treatment commenced, it should have been above natural; and for the first few days of treatment she should have lost weight.

Now, what did the extra oxygen do? Brought the rate, or velocity of repair and waste up to their normal standard. Constructive and destructive metamorphoses were both quickened, and her thermal condition normal, or slightly above, and steady.

While the excess of oxygen was successful in relieving this invalid in less than a month, it was, probably, by no means the only treatment or means by which she might have attained the same ends. Forced exercise in the open air—hard work, if you please—a visit to the sea-shore, or some saline springs, would have, in all probability, as certainly relieved her, and very likely quite as rapidly, because they would do, in the end, precisely what the excess of oxygen did, viz., bring the organic processes of waste and repair up to their normal standard. "Up to their normal standard" is written understandingly, for no diseased action is above or higher than life. No matter how *high inflammation may run*, in any given case, it will always be found a lower grade than normal of organic life, tending, not to higher life, but to death.

Case 2d.—This patient, a lady, was probably, too, from the "well-to-do" class of people, as well as No. 1. The same abnormal phenomena prominent in both. Neither constructive nor destructive metamorphoses were proceeding normally in her case. The excess of free oxygen given in the commencement of the treatment set up a too rapid waste, not in the region of the kidneys alone, but over the whole system, and it had to be greatly diminished for a few days, and some measures resorted to to retard it. At the end of something over five weeks, a chalybeate was added to the oxygen treatment to promote constructive metamorphosis, and when the processes of nutrition and waste were restored to their normal velocity, she, too, returned to her friends, greatly benefited.

In her case, compulsory physical exercise in the open air, combined with a sulphur and chalybeate course of natural mineral waters, would, in all probability, have accomplished the same ends.

Case 3d.—From the same "well-to-do" class as the first and second; the cause of her trouble the same. Not sufficient labor to keep constructive and destructive metamorphosis proceeding at normal velocity. No structural lesions, and, as a consequence, the super supply of oxygen performed its mission with certainty. In something over two weeks they were brought up to their normal velocity, and she, too, recovered.

Exercise in the open air, the sea-shore, or saline springs, would have done the same thing.

Case 4th.—So nearly like cases one, two and three as not to require a repetition of the remarks applicable to them all.

Case 5th.—More complicated, and in a male; but still from the "well-to-do" class. The rate of destructive metamorphosis being too tardy, and elimination imperfect, of course he is a "martyr" to "the dyspepsy." Surely, in his case the lesion is in the altered relations of nutrition and oxidation. He inhales the super supply of oxygen, it burns up his effete tissues, and the debris of tissue metamorphosis, retained in his system, is eliminated; and, as soon as the processes of constructive and destructive metamorphosis are returned to their normal velocity, he, too, is well.

Three months fishing off New Foundland, or a sea-voyage, or a season's work in the turpentine business in the Carolinas, would have accomplished the same ends for him.

Let me say, again, no fault is found with the treatment pursued in these cases by Dr. Wallihan; on the contrary, much credit is due him for his discrimination and success; but his treatment was, after all, empirical; he had, or at least gives, no philosophy for the mode of operation of the excess of oxygen employed in the treatment of them; but here is a formula that will satisfactorily explain the whole thing:

"Super oxygenation compensates for deficiency of physical exercise or labor, in restoring the normal velocity of tissue-changes, or "constructive and destructive metamorphosis."

Has the purpose for which the paper was written been accomplished? Cannot purpose, means and results in the treatment of the whole catalogue of human maladies be more definitely connected by regarding all diseased action as modifications of the organic processes of nutrition and oxidation, and all therapeutic agencies as promoting or retarding them, than in any other way?

Reference to other therapeutic measures than super oxygenation was purposely avoided in commenting on the salient features of them. Only natural agencies were pointed out. It is very probable they all could have been successfully treated without the direct excess of oxygen in the mode administered. It is very certain that such cases have been successfully treated in my practice by using salines, iodides and bromides, to promote oxidation, with metallic oxides and vegetable bitters—the metallic oxides more particularly in "hysterical" or painful cases, or lesions of force, and simple bitters and iron for the more prominent lesions of mutation—to promote nutrition, and there is no incompatibility or failure in using both classes of agents at the same time. But "well-to-do" people will not work in the open air, nor visit saline springs, nor go fishing off New Foundland, so it is a happy circumstance that super-oxygenation enables us, as well as with other means, to relieve them from enduring the penalties inflicted on them for a violation of the laws of organic life.

